# Effect of Nb on Microstructure and Mechanical Property of Novel Powder Metallurgy Superalloys during Long-Term Thermal Exposure

**DOI:** 10.3390/ma14030656

**Published:** 2021-01-31

**Authors:** Dingmao Zhou, Xianjue Ye, Jianwei Teng, Chao Li, Yunping Li

**Affiliations:** State Key Lab for Powder Metallurgy, Central South University, Changsha 410083, China; Tracy_zdm@csu.edu.cn (D.Z.); yexianjue@csu.edu.cn (X.Y.); tengjianwei@csu.edu.cn (J.T.); Lichao1997@csu.edu.cn (C.L.)

**Keywords:** superalloy, Nb, thermal exposure, γ′ coarsening, Vickers hardness, strengthening mechanisms

## Abstract

Microstructure and mechanical properties of novel Ni-20Co-12Cr superalloys, with and without Nb addition, were systematically studied during long-term thermal exposure. With increased exposure time, the average diameter of the γ′ precipitates increased in both alloys in succession; this is more obviously observed in alloy containing 1 wt% Nb (1Nb). It is suggested that Nb increased the γ′ coarsening rate by accelerating the diffusion of Al and Nb in γ matrix. In addition, the γ′ phase fraction is increased by about 4% in 1Nb compared to the alloy without Nb (0Nb). The morphology of the γ′ phase changed from near-spherical to cuboidal shape during exposure in both alloys. Due to the increased γ/γ′ lattice misfit by Nb addition, 1Nb alloy showed an earlier tendency of shape change. Vickers hardness results revealed that the overall hardness decreased with the exposure time because the size increment of the γ′ precipitate weakened the precipitates strengthening and Orowan strengthening.

## 1. Introduction

Powder metallurgy (PM) nickel-based superalloys demonstrate high fatigue resistance, hot-corrosion resistance, high tensile and stress-rupture properties, as well as high oxidation resistance up to 900 °C, and have been widely used as turbine disc materials in aero-engines and power generation turbines [1,2,3]. With increasing operation temperature in new generation aeroengines, novel superalloys with higher mechanical properties and higher oxidation resistance are urgently required [4,5]. 

The typical microstructure of nickel-based superalloys includes disordered γ matrix and ordered γ′-Ni_3_(Al, Ti, Nb) precipitates. The face-centered-cubic (A_1_) γ matrix is strengthened by a dispersion of coherent intermetallic γ′ precipitates with an L_12_ crystal structure [1]. PM nickel-based superalloys usually consist of more than 10 elements. Among them, Al, Ti, Ta, and Nb are known as the γ′ forming elements. Co, W, Cr, Mo are known as solid solution strengthening elements. Different atom radii between Ni and solutes cause lattice mismatch and strain field, which have elastic interaction with dislocation [6] and contribute to the γ matrix’s strength increment. C, B, Zr, Hf are grain boundary strengthening elements as they can form fine carbides and borides, which preferentially precipitate at grain boundaries (GBs) and sub-boundary, improving the cohesion of boundaries and effectively preventing crack initiation [7,8]. 

During service, superalloys usually undergo various microstructural changes including coarsening of γ′, formation of topologically close-packed (TCP) phase and continuous carbide network along GBs, as well as degeneration of MC carbides [9,10,11]. These microstructure changes pose different effects on mechanical properties. The mechanical behaviors of nickel-based superalloys are affected by γ′ size distribution and volume fraction consumingly [12]. Both of them evolve significantly with exposure temperature or time because of the dissolution of tertiary γ′ precipitates and redistribution of alloying elements under non-equilibrium conditions [2]. Higher working temperature means severer microstructure change. Hence, in order to develop PM nickel-based superalloys, it is important to figure out the role of elements and environmental effect on the microstructure and mechanical performance during service.

Nb additions will increase the γ′ content and play a role as the potential solid solution strengthening element in the γ matrix [6]. Nb can replace Al and Ti in γ′, and it decreases the solution of Al and Ti in the γ matrix, leading to more γ′ formation [13]. Furthermore, Nb substituting onto the Al sublattice increased the anti-phase boundary (APB) energy of the γ′ phase [14,15], further contributing to the strength. Recently, Christofidou et al. [16] studied the effect of Nb on the oxidation behavior and mechanical properties of next generation polycrystalline PM nickel-based superalloys, finding that Nb increased the volume fraction of γ′ precipitates, which could lead to superior oxidation resistance as well as high tensile and creep properties. Nevertheless, high Nb concentrations have been related to the precipitate of the η (Ni_3_Ti) and δ (Ni_3_Nb) phases, as well as the formation of σ phase, which are generally considered deleterious to mechanical properties [17,18,19]. Besides, Nb strongly affects the carbides’ formation and distribution. Wang et al. [20] investigated the effect of different Nb contents on the carbides of Ni-12Mo-7Cr-based cast superalloys during long-term thermal exposure, showing that high Nb content (4 wt.%) leads to NbC carbides formation as well as different interface structures and orientation relationships between carbides and γ matrix. However, in the PM nickel-based superalloys, although much effort has been made to study the influence of Nb on the microstructure and mechanical properties, the effect of Nb addition on γ′ stability and its relationship with mechanical properties changing during long-term thermal exposure is less well known.

Therefore, in this work, Ni-20Co-12Cr-based PM superalloys without and with 1 wt% Nb were prepared. In order to simulate the working condition, specimens were thermal exposed at 800 °C, which is the critical service temperature of turbine disks. After that, the volume fraction and size of the γ′ precipitates were systematically characterized. The coarsening kinetic of γ′ during long-term thermal exposure was also discussed. Meanwhile, to connect microstructural evolution with mechanical property change, the hardness of specimens at different exposure times was tested and different strengthening mechanisms contributing to Vickers hardness were discussed in detail.

## 2. Experimental Procedure

Novel polycrystalline Ni-20Co-12Cr superalloys with different Nb concentrations (0% and 1 wt%) were produced by powder metallurgy (PM) processing route. The alloy compositions tested by inductively coupled plasma optical emission spectroscopy (ICP-OES) are shown in Table 1. The argon atomization (AA) powders of the 0Nb and 1Nb alloys were sieved to a final screen size of 150 mesh (under 100 µm) and filled into mild steel containers and then hot isostatically pressed (HIP) under 1180 °C/150 MPa for 4 h. The densities of the 0Nb and 1Nb alloys after HIP were measured to be 8.45 and 8.46 g/cm^3^, respectively, which are extremely close to their theoretical densities. Long-term thermal exposure specimens (10 mm × 10 mm × 2 mm) were cut from the HIP ingots. After that, standard heat treatment (SHT) for specimens was performed: solid solution at 1180 °C (above the γ′ solvus temperature) for 2 h followed by air cooling and then aged at 800 °C for 16 h followed by air cooling. After SHT, specimens were exposed in air at 800 °C up to 1000 h.

Change of microstructure owing to thermal exposure was characterized by field emission scanning electron microscopy (SEM; FEI Quanta 650 FEG, Brno, Czech Republic). The metallographic specimens were prepared by standard metallographic techniques. In order to measure the volume fraction and size of γ′, a chemical etch (for 10 s in a solution of 33% H_2_O + 33% Acetic acid + 33% HNO_3_ + 1% HF) and an electrical etch (at 5 V for 10 s in a solution of 40% H_2_SO_4_ + 12% H_3_PO_4_ + 48% HNO_3_) were conducted correspondingly. The reason for adoption of different methods was that the γ matrix was selectively etched out by chemical etch which could bring an overestimation of γ′ size while the γ′ was selectively etched out by electrical etch which could induce an overestimation of the γ′ fraction [21]. 

With the SEM micrographs, the volume fraction and size of γ′, which are described as area percentage and equivalent circular diameter, respectively, were quantitatively measured by using image analysis software (Fiji distribution of ImageJ 2.1.0 [22]). In addition, the grain size distributions of alloys were measured by the electron backscatter diffraction (EBSD) technique, which was performed with an Oxford Instruments AZtec system (Abingdon, UK). 

Furthermore, the equilibrium chemical compositions of γ and γ′ were calculated at 800 °C by Thermo-Calc software with TTNI8 database (Thermotech, Surrey, UK). The diffusion coefficients in γ matrix of different elements were calculated by DICTRA with TTNI8 and MOBNI1 database.

The Vickers hardness of each sample was tested after exposed for different time using a Vickers hardness tester (THV-10, TEST-TECH Co., Ltd, Shanghai, China). The surfaces of samples were firstly ground with fine SiC paper of #2000. During the test, samples were loaded up to the peak load of 2 kg under a dwell time of 10 s. Each value was taken as an average of ten measurements.

## 3. Results 

**Figure 1:**
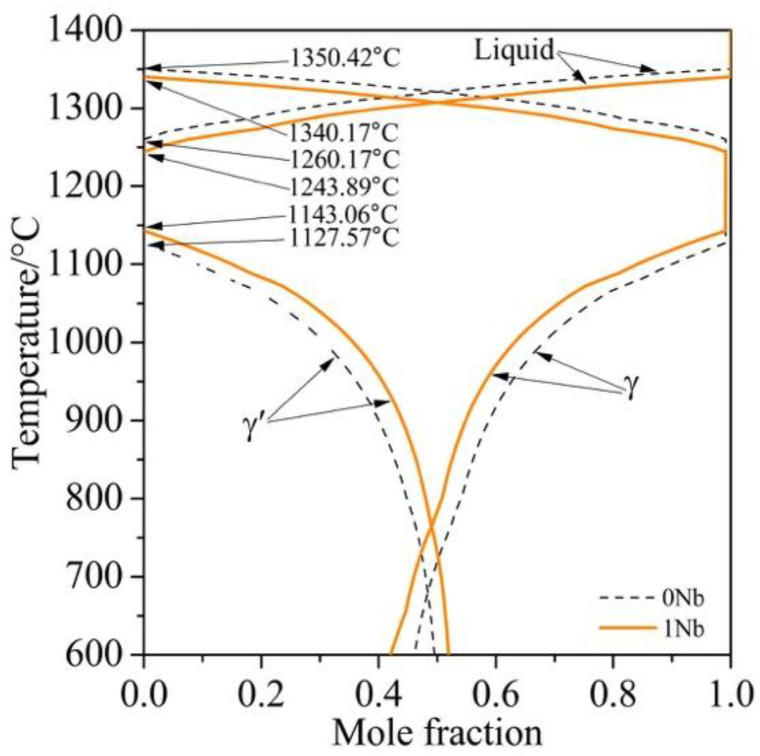
Phase diagram of the 0Nb and 1Nb alloys calculated by Thermo-Calc with TTNI8 database.

The phase diagrams of the 0Nb and 1Nb alloys calculated by Thermo-Calc with TTNI8 database are shown in Figure 1, which provides the information on the equilibrium phase fraction present in the alloy at different temperatures. The γ′ solvus temperatures of the 0Nb and 1Nb alloys are 1127.57 °C and 1143.06 °C, respectively, implying the increased driving force of γ′ phase formation by Nb addition.

**Figure 2:**
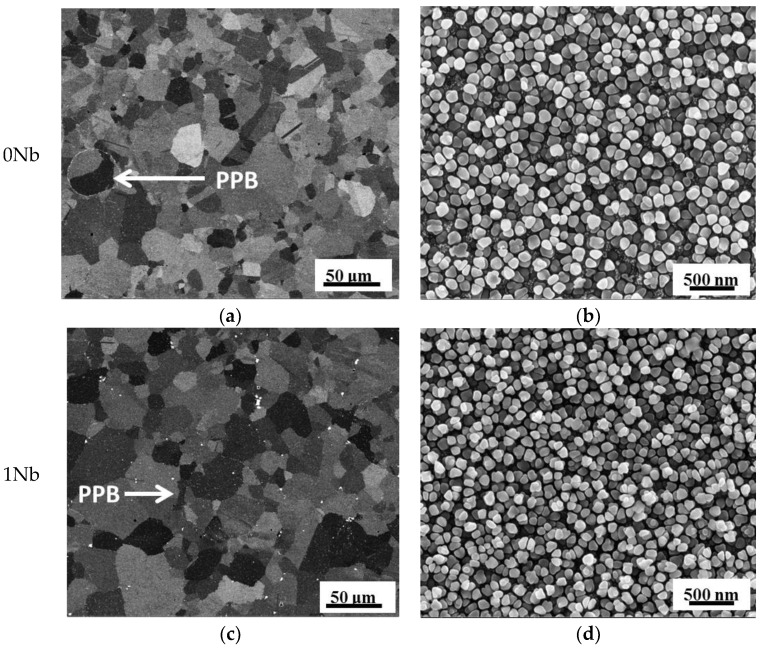
Microstructure of the 0Nb (**a,b**) and 1Nb (**c,d**) alloys after standard heat treatment (SHT) (**a,c**) backscatter electron (BSE) images; (**b,d**) second electron (SE) images.

The microstructures of the 0Nb and 1Nb alloys after SHT are shown in Figure 2. The backscatter electron (BSE) images (Figure 2a,c) show that both alloys exhibit comparable grain size of about 10 μm. Additionally, prior particle boundaries (PPBs) exist in both alloys. The PPBs resulted from powder surface contamination, which was originated from solidification segregation and surface adsorption [23]. The second electron (SE) images after electrical etch, Figure 2b,d, reveal that the γ′ morphology of the 0Nb and 1Nb alloys present uniformed size and near-spherical shape. Notably, no TCP phase is observed in both alloys.

**Figure 3:**
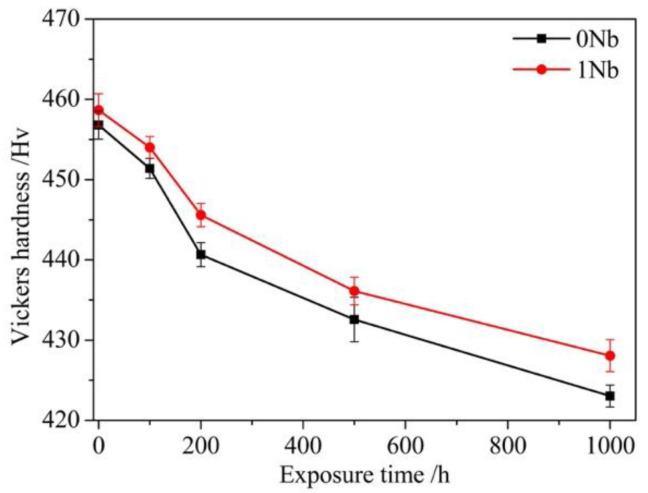
Vickers hardness of the 0Nb and 1Nb alloys during long-term exposure at 800 °C up to 1000 h.

Vickers hardness was used for investigating the exposure-induced variation in the hardness. Figure 3 shows the results that the hardness values of the 0Nb and 1Nb alloys were 456.8 and 458.7 HV correspondingly at the beginning. With exposure time increased, both alloys exhibited the declined hardness. The Vickers hardness decreased obviously between 0 h and 200 h, and then turned to relatively mildly decrease between 200 h and 1000 h. After exposure at 800 °C for 1000 h, the hardness of the 0Nb and 1Nb alloys was 423 and 428 HV, respectively. In addition, the hardness of the 1Nb alloy maintained higher than that of the 0Nb alloy throughout the exposure.

**Figure 4:**
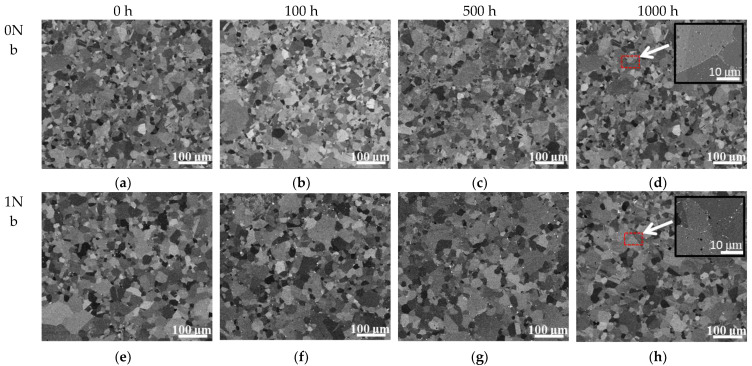
(**a**–**h**) Backscatter electron (BSE) images of the 0Nb and 1Nb alloys during long-term exposure at 800 °C.

The morphologies of the 0Nb and 1Nb alloys exposed at 800 °C in different time are shown in Figure 4. These results clearly indicate that the grain size differs little throughout the exposure in both alloys. In addition, the formation of discontinuous carbides at the grain boundaries was observed in both alloys, which can be seen in the partially enlarged detail in Figure 4d,h.

**Figure 5:**
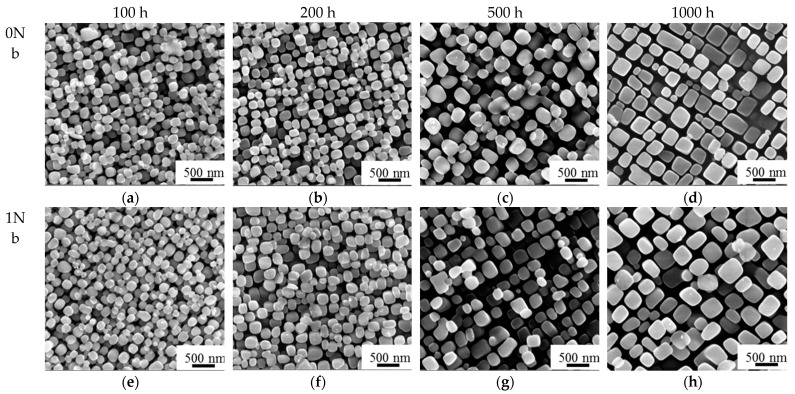
(**a**–**h**) SEM morphology of γ′ of 0Nb and 1Nb during long-term exposure at 800 °C.

Figure 5 shows the morphology of the γ′ precipitates during long-term exposure in different times. It can be seen that the γ′ precipitate of the 0Nb and 1Nb alloys grew larger gradually with exposure time increasing. The particle shapes in two alloys also changed from near-spherical to cuboidal shape gradually, of which the 1Nb alloy showed an earlier tendency of this transition. The shape of the γ′ precipitates exposed for 500 h in 1Nb alloy was more cuboidal than that in 0Nb alloy.

**Figure 6:**
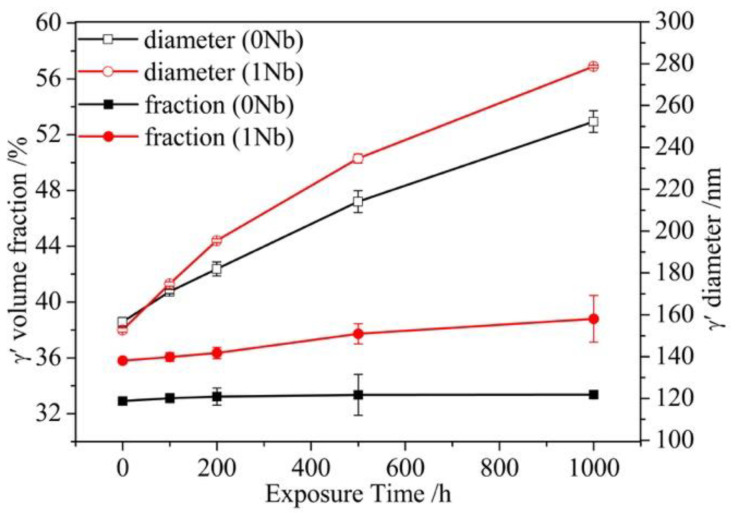
γ′ size and volume fraction evolution during long-term thermal exposure at 800 °C up to 1000 h.

Volume fraction and size of γ′ were quantitatively analyzed using high-resolution SEM images. For each exposure time, at least six micrographs were randomly selected and used to measure the γ′ fraction and γ′ phase particle size. The results shown in Figure 6 suggest that with increasing exposure time, the average γ′ size of two alloys increased constantly where faster increment occurred in 1Nb alloy. Meanwhile, the γ′ volume fraction of the 1Nb alloy increased slightly while that of the 0Nb alloy was almost a constant. The γ′ content of the 1Nb alloy is about 4% higher than that of 0Nb, as Nb replaced Al and Ti in γ′ and decreased the solution of Al and Ti in the γ matrix, which facilitated the formation of γ′ [13].

## 4. Discussion

### 4.1. Microstructural Evolution

#### 4.1.1. Grain Size Evolution during Exposure

**Figure 7:**
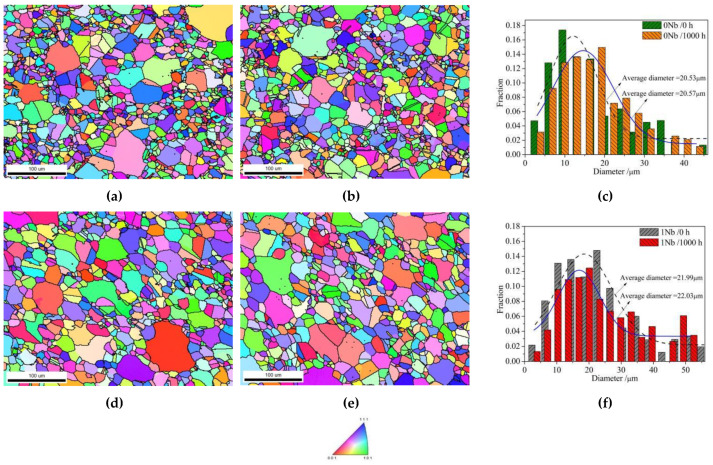
Inverse polar figure (IPF) of (**a**) 0Nb/0 h; (**b**) 0Nb/1000 h; (**d**) 1Nb/0 h; (**e**) 1Nb/1000 h; grain size distribution of (**c**) 0Nb; (**f**) 1Nb.

From the aforementioned results, it can be seen that both 0Nb and 1Nb alloys consist of large and small grains. The particle size distributions of the alloy powders were in the range of 0–100 μm so that the grains were restricted inside the powder particles. Therefore, grains grow takes place inside the particles, resulting in inhomogeneous grain size. In addition, there is no obvious change in the average grain size during long-term exposure. To confirm this, inverse polar figures (IPFs) of samples at 0 h and 1000 h were used for counting the grain size. The results in Figure 7 show that, as the exposure time increases from 0 h to 1000 h, the average grain size of the 0Nb alloy changes from 20.53 to 20.57 µm. Meanwhile, that of the 1Nb alloy also increases slightly, from 21.99 to 22.03 µm. The results indicate that the grain size in both alloys did not change after long-term exposure. This could be owing to the formation of discontinuous carbide precipitates at grain boundaries. The discontinuous carbides nailed onto the grain boundaries, which hindered the grains’ growth.

#### 4.1.2. Shape Change of γ′ Precipitates

The shapes of γ′ in the two alloys changed from near-spherical to the cuboidal shape gradually where the transformation may be ascribed to the γ/γ′ lattice misfit at exposure temperature [24]. The γ′ precipitates are spherical when the lattice mismatch is closed to zero, for instance, in the Ni-Cr-Al alloys, whereas the shape of the γ′ precipitates is rod-like or plate-like when the lattice mismatch is large, for example, in the Ni-Be alloys. The γ′ precipitates are cubes in a row in the alloy system with intermediate lattice mismatch, such as Ni-Al and Ni-Si. Moreover, when the lattice mismatch is large enough, the rows will sometimes coalesce into rods [24]. 

The lattice misfit is determined by the lattice parameters a_i_ of the phases, which are controlled by the chemical composition of the phases at the exposure temperature via Vegard’s law and by the heat expansion coefficients of both phases [25]. The misfit is defined as:

(1) $$\delta =\frac{2\left(a_{\gamma^{\prime }}-a_{\gamma }\right)}{a_{\gamma^{\prime }}+a_{\gamma }}$$

where a_γ_ and a_γ′_ are lattice parameters of the γ matrix and γ′ precipitates, respectively. According to Caron [26], the lattice parameters (Å) of the γ and γ′ phase at room temperature can be given by:

(2) $$a_{\gamma }^{\mathrm{RT}}=a_{\mathrm{Ni}}+\sum^{\;}V_{i}x_{i}$$

(3) $$a_{\gamma^{\prime }}^{\mathrm{RT}}=a_{{\mathrm{Ni}}_{3}\mathrm{Al}}+\sum^{\;}V_{i}^{\prime }x_{i}^{\prime }$$

where a_Ni_ and $a_{{\mathrm{Ni}}_{3}\mathrm{Al}}$ corresponding to the lattice parameters of Ni and Ni_3_Al, were taken as 3.524 Å and 3.570 Å correspondingly [26]; X_i_ and $x_{i}^{\prime }$ mean the mole fraction of element i in γ and γ′ phase at 800 °C, respectively; V_i_ and $V_{i}^{\prime }$ represent the Vegard coefficient for element i in Ni and Ni_3_Al, respectively. In addition, the misfit is strongly influenced by temperature. Therefore, in order to indicate the different thermal expansion of γ and γ′ phase, the method from Caron is extended by containing the heat expansion coefficients for pure Ni and Ni_3_Al by Kamara [27]. The lattice parameters (Å) considering thermal expansion at relevant temperature can be calculated by:

(4) $$a_{\gamma }=a_{\gamma }^{\mathrm{RT}}+5.74\times 10^{-5}\times T-1.010\times 10^{-9}\times T^{2}$$

(5) $$a_{\gamma^{\prime }}=a_{\gamma^{\prime }}^{\mathrm{RT}}+6.162\times 10^{-5}\times T-1.132\times 10^{-8}\times T^{2}$$

where T is absolute temperature. The mole fraction (calculated by Thermo-Calc software with the TTNI8 database) and Vegard coefficients (from reference [26]) of element *i* in γ and γ′ phase are listed in Table 2. The calculated lattice parameters and misfit *δ* of the 0Nb and 1Nb alloys at 800 °C are listed in Table 3. One can see that the addition of Nb enlarged the γ/γ′ lattice misfit. When the magnitude of the misfit is small, the γ′ particles have to grow to a larger size before the cuboidal form is found [1]. In terms of the 0Nb alloy, the precipitate phase needed a longer exposure time to coarsen to a larger size before its shape changes. So, this can explain why the shape change of γ′ took place earlier in the 1Nb alloy. In essence, Nb addition promoted the shape change from near-spherical to cuboidal shape. Nevertheless, the shape change in both alloys was not significant compared with other nickel-based superalloys [28,29,30,31,32], and this relatively higher stability of γ′ precipitates is good for the mechanical properties during high-temperature service.

#### 4.1.3. γ′ Coarsening

During the thermal exposure process of the Ni-based superalloys, the content and size of γ′ increased gradually. The amount of γ′ finally approached to a constant when aging finished. In the later exposure period, big size γ′ kept growing with small γ′ dissolved gradually, which was driven by interfacial energy [35]. As a result, the average size of γ′ increased while the amount decreased. This is so-called γ′ coarsening. 

In the elastic constraint-free or weakly constraint systems, which have relatively small lattice mismatch, γ′ coarsening is controlled by diffusion and following the third power law [28,36]. In strongly constrained systems, the lattice mismatch is large, and the elastic effect plays a dominant role [24]. In this work, the coarsening of the γ′ precipitates during thermal exposure was controlled by diffusion, which could be analyzed by Lifshitz–Slyozov–Wagner (LSW) theory [37,38]. The growth kinetics would follow the third power law given as:

(6) $$r_{t}^{3}-r_{0}^{3}=\mathrm{kt}$$

where r_t_ represent the averaged radius of γ′ precipitates at different time t, r_0_ is the original radius taken from the SHT sample, and k is the rate constant of coarsening, given as:

(7) $$k=\frac{8{\sigma \mathrm{DV}}_{m}C_{m}}{9\mathrm{RT}}$$

where σ is the interfacial energy between precipitates and matrix, D is the diffusion coefficients of solute atoms in the γ matrix, C_m_ is the concentration of solute in the matrix in equilibrium with an infinitely large precipitate, V_m_ is the molar volume of the precipitates, R is the Avogadro constant, T is the absolute temperature.

**Figure 8:**
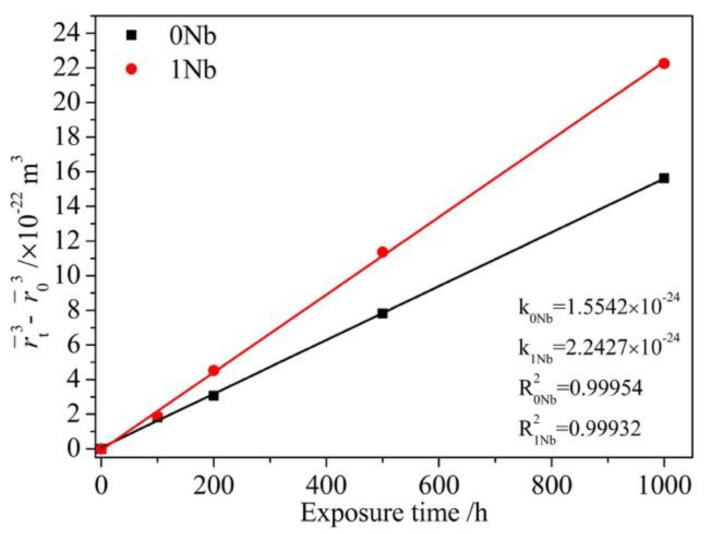
Lifshitz–Slyozov–Wagner (LSW) analysis for γ′ coarsening.

In Figure 8, it is seen that the linear fit for the averaged radius of the precipitates vs. time in two alloys greatly followed the relation in Equation (6). The coarsen rate of the 1Nb alloy is higher than that of the 0Nb alloy. According to Equation (7), for a given temperature, R and T are constant. V_m_ is only related to the lattice constant of the γ′ phase. As shown in Table 3, the addition of Nb showed no significant influence on the γ′ lattice constant so that the variation of the quantity V_m_ with Nb content can be neglected. The interfacial energy σ is associated with the structure of the γ/γ′ interface and the chemistry of interface [39]. Both of them rarely change with Nb content. Therefore, σ and V_m_ are taken as constant for the two alloys at same temperature. However, C_m_ declined slightly with Nb content as shown in Table 2, which decreased the coarsening rate k according to Equation (7). By reasons of the foregoing, the higher coarsening rate in the 1Nb alloy probably came from the discrepancy of diffusion coefficient between two alloys. Therefore, the influence of Nb addition in the diffusion constants in γ matrix of solute atoms was calculated. ΔD is defined as the increment of diffusion coefficient with Nb addition, and D_0_ is the diffusion coefficient without Nb. ΔD is given as:

(8) $$\Delta D=D-D_{0}$$

**Figure 9:**
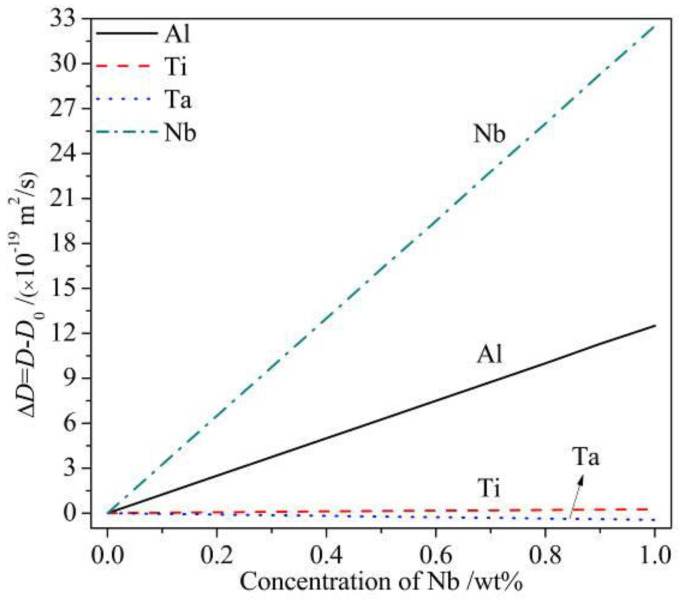
Diffusion coefficient increment of Al, Ti, Ta, Nb in γ matrix at 800 °C with different Nb concentration (calculated by DICTRA with the TTNI8 and MOBNI1 database).

As shown in Table 2, element Al, Ti, Ta, Nb mainly portioned in γ′ phase, played a role as γ′ former. The diffusion coefficients in γ matrix at 800 °C of these elements were calculated by DICTRA software in TTNI8 and MOBNI1 databases. The results in Figure 9 show that, with Nb addition increased, the diffusion coefficients of Ti and Ta slightly decreased while that of Al and Nb increased greatly, which indicated that Nb accelerated the Al and Nb atoms moving to big size γ′ from the γ matrix. The particle radius changed with diffusion of solute atoms which flowed into or out of the particle. Therefore, Nb addition accounts for the higher coarsening rate and the faster γ′ growth in 1Nb alloy.

### 4.2. Mechanical Property Variation

Vickers hardness of two alloys was found to decrease during exposure. In order to figure out how the Nb addition and thermal exposure affected the hardness variation, the extent of different strengthening mechanisms to the total yield strength are discussed below, including precipitate strengthening, solid solution strengthening, grain boundaries strengthening and Orowan strengthening. 

According to Wang [40] and Wu [41], the yield strength is positively related to the Vickers hardness for nickel-based superalloys:

(9) $$\sigma_{y}=2.34H_{v}$$

Therefore, the Vickers hardness of the 0Nb and 1Nb alloys exposed for different time in this experiment can be converted to yield strength $\sigma_{y}^{\exp}$ by Equation (9).

#### 4.2.1. Precipitate Strengthening

The strengthening of precipitates in superalloys is usually governed by the cutting mechanism which can be divided into two models for small precipitates and large precipitates, namely, the weakly coupled dislocation (WCD) model and the strongly coupled dislocation (SCD) model correspondingly. Cutting mechanism leading to precipitate strengthening was evaluated in the light of the critical resolved shear stress (CRSS). Based on assuming a pair of edge dislocations slip in the [1 1 0] direction on the {1 1 1} plane and cut through the ordered γ′ precipitates in a disordered matrix, one can estimate the CRSS necessary for cutting the precipitates for both cases [42].

In so far as the WCD model is applicable to fine precipitates, the CRSS can be calculated by:

(10) $$\tau_{p,\mathrm{WCD}}=\frac{1}{2}{\left(\frac{\gamma_{\mathrm{APB}}}{b}\right)}^{\frac{3}{2}}{\left(\frac{\mathrm{bdf}}{T}\right)}^{\frac{1}{2}}A-\frac{1}{2}\left(\frac{\gamma_{\mathrm{APB}}}{b}\right)f$$

where d is the precipitate diameter, T is the line tension of the dislocation, b represents the Burgers vector of edge dislocation in the matrix, f means the volume fraction of the γ′ precipitates, γ_APB_ is the anti-phase boundary energy (APBE) of γ′ in the {1 1 1} plane, and A is numerical factor determined by the γ′ morphology (which is 0.72 for spherical precipitates).

Regarding the SCD model account for larger precipitates, the CRSS can be described as:

(11) $$\tau_{p,\mathrm{SCD}}=\left(\frac{\sqrt{3}}{2}\right)\frac{{\mathrm{wTf}}^{\frac{1}{2}}}{\mathrm{bd}}{\left(1.28\frac{{d\gamma }_{\mathrm{APB}}}{\mathrm{wT}}-1\right)}^{\frac{1}{2}}$$

where the values of b and γ_APB_ were taken as 0.254 nm and 0.275 J/m^2^, respectively [42], w is a constant (with an order of unity) that explains the elastic repulsion between SCD and is taken as 1 in this paper. The line tension T was estimated as 0.5 Gb^2^ [43] and the shear modulus G was taken as 80 GPa [42].

**Figure 10:**
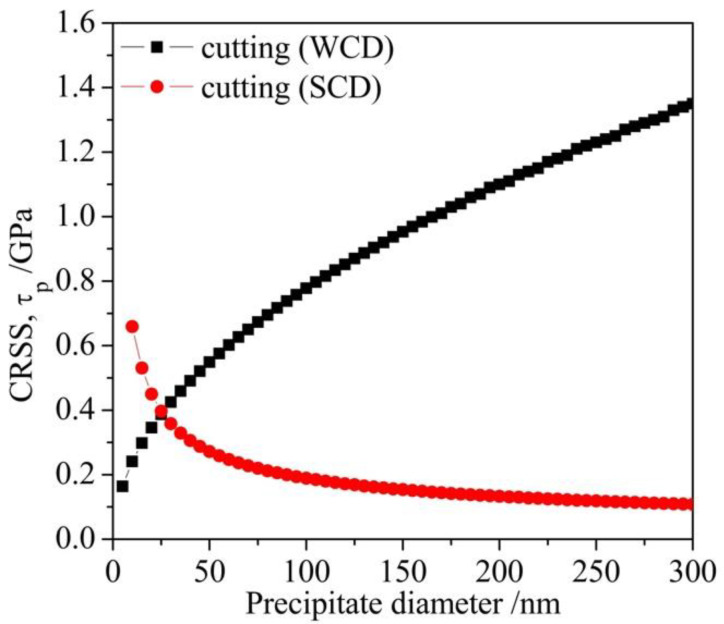
Analytical calculations of critical resolved shear stress (CRSS) vs. precipitate diameter including weakly coupled dislocation (WCD) model and the strongly coupled dislocation (SCD) model.

The contribution of different cutting mechanism to the overall Vickers hardness can be estimated with the values of the CRSS calculated according to Equations (10) and (11). Figure 10 shows the calculated CRSS for different cutting mechanisms (τ_p_) that were drawn against the precipitate diameter. The content of precipitates was taken as the average values for each sample (as seen in Figure 6) in this theoretical calculation. As shown in Figure 10, the mechanism with lower CRSS mainly contributed to the strengthening for a given diameter. With the increasing size of the precipitate, the governing mechanism switches from the WCD mechanism to SCD cutting. Therefore, it can be seen in Figure 6 that, as the average diameter of γ′ is more than 100 nm in the present work, the strength of the two alloys is mainly contributed by the SCD mechanism. The shear stress τ_p,SCD_ can be transformed to a normal stress σ_p_ by multiplying the Taylor factor M (taken as 3) [33].

(12) $$\sigma_{p}={M\tau }_{p,\mathrm{SCD}}$$

#### 4.2.2. Solid Solution Strengthening

According to Labusch [44] theory, solid solution hardening is estimated. The solute atoms act as frictional barriers for dislocation slip in a binary alloy leading to improvement in the yield stress. This is governed by the change of modulus and local lattice in the solid solution. This approach was modified by Gypen and Deruytterre [45,46] to aggregate strength increment of different alloying elements in multicomponent systems. Atomic size and modulus of solute elements are disparate in the γ and γ′ phases. The degree of solid solution strengthening ($S_{i}^{\gamma }$) in the disordered γ matrix phase can be calculated as [33]:

(13) $$S_{i}^{\gamma }=\beta_{i}^{\gamma }{\left(x_{i}^{\gamma }\right)}^{\frac{1}{2}}$$

where $x_{i}^{\gamma }$ means the concentration of element i in the matrix. The constant $\beta_{i}^{\gamma }$ given in Table 2 is the strengthening coefficient for the matrix (from reference [33,34]) associated with modulus and atom radius, indicating the strengthening effects of different solid solution elements. 

Different tendencies in atomic bonding between neighboring atoms result in disparate solid solution strengthening in the ordered γ′ phase as well [47]. This can be adjusted by changing the exponent of the resulting strength equation [47,48]:

(14) $$S_{i}^{\gamma^{\prime }}=\beta_{i}^{\gamma^{\prime }}x_{i}^{\gamma^{\prime }}$$

where $x_{i}^{\gamma^{\prime }}$ represents the concentration of element i in the γ′ phase and the constant $\beta_{i}^{\gamma^{\prime }}$ is the strengthening coefficient for γ′, which is also given in Table 2.

The contributions of each element can be integrated to confirm the total influence of solution strengthening. [46]. The volume fraction of each phase was considered in the calculation, given as:

(15) $$\sigma_{\mathrm{sss}}^{\gamma }=\left(1-f_{\gamma^{\prime }}\right){\left[\underset{i}{\sum }{\left(S_{i}^{\gamma }\right)}^{2}\right]}^{\frac{1}{2}}$$

(16) $$\sigma_{\mathrm{sss}}^{\gamma \prime }=f_{\gamma^{\prime }}\underset{i}{\sum }S_{i}^{\gamma^{\prime }}$$

where f_γ′_ is the volume fraction of the γ′ precipitates obtained experimentally (see Figure 6). The total solid solution strength was given as:

(17) $$\sigma_{\mathrm{sss}}=\sigma_{\mathrm{sss}}^{\gamma }+\sigma_{\mathrm{sss}}^{\gamma^{\prime }}$$

#### 4.2.3. Grain Boundary Strengthening

The extent of grain boundary strengthening is well summarized by the Hall–Petch relation:

(18) $$\sigma_{\mathrm{HP}}=\frac{k_{\mathrm{HP}}}{\sqrt{D}}$$

where D represents the average grain size listed in Table 4, k_HP_ is a constant taken as 750 MPa/μm^−1/2^ (which is in the range of 710–750 MPa /μm^−1/2^ for superalloys [49]).

#### 4.2.4. Orowan Strengthening

Large precipitates in the γ matrix may be bypassed by Orowan looping at some conditions. The contribution in strength resulted from this mechanism, σ_Oro_, can commonly be calculated by [21,49]:

(19) $$\sigma_{\mathrm{Oro}}=M\frac{\mathrm{Gb}}{\lambda }$$

where the shear stress is transformed to a normal by multiplying the Taylor factor M (taken as 3). The λ is inter-particle spacing between precipitates and is often simplified as [21]:

(20) $$\lambda =\frac{2\left(1-f_{\gamma^{\prime }}\right)d}{3f_{\gamma^{\prime }}}$$

#### 4.2.5. Yield Strength

The yield stress σ_y_ in superalloys includes four strengthening contributions [50]: (i) precipitate strengthening (σ_p_); (ii) solid solution strengthening (σ_sss_); (iii) grain boundary strengthening (σ_HP_); (iv) Orowan strengthening (σ_Oro_):

(21) $$\sigma_{y}=\sigma_{p}+\sigma_{\mathrm{sss}}+\sigma_{\mathrm{HP}}+\sigma_{\mathrm{Oro}}$$

**Figure 11:**
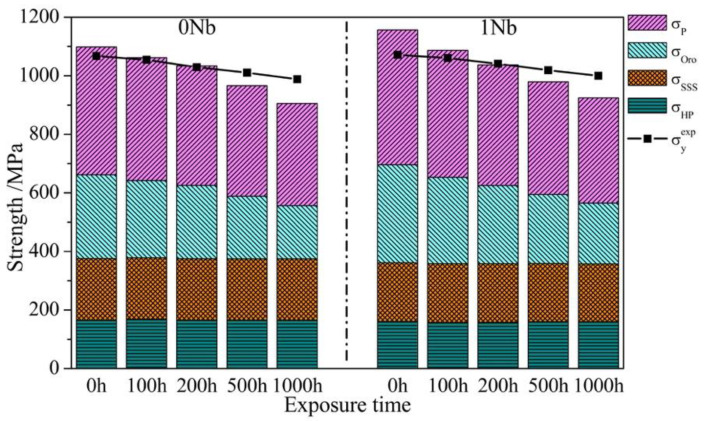
Extent of each strengthening mechanisms to the yield strength at ambient temperature in 0Nb and 1Nb alloys where all samples were exposed at 800 °C.

$\sigma_{y}^{\exp}$ and calculated σ_y_ contributed from the individual strengthening mechanisms are shown in Figure 11. The calculated values σ_y_ greatly match the experimental values $\sigma_{y}^{\exp}$. It can be seen that the precipitate strengthening makes the greatest contribution to the yield strength. The Orowan strengthening is another strengthening mechanism from the γ′ precipitates, which made non-negligible enhancement to σ_y_ as well. In addition, solid solution strengthening and grain boundary strengthening are the other two major factors contributing to the yield strength at ambient temperature. After thermal exposure, σ_HP_ and σ_sss_ were almost unchanged, while σ_Oro_ and σ_p_ declined obviously. This was because γ′ precipitates coarsened during exposure, while Orowan bowing strengthening and SCD cutting strengthening are strongly dependent on the γ′ size. According to Figure 10 and Equations (19) and (20), σ_Oro_ and σ_p_ will decrease with the mean diameter d of γ′ increasing. Evidently, the extent of σ_y_ decreased with exposure time increasing, which was mainly resulted by the reduction in σ_Oro_ and σ_p_. Therefore, the reduction in Vickers hardness can be ascribed to γ′ precipitates coarsening during long-term exposure.

With regard to the Vickers hardness of the 1Nb alloy being larger than that of the 0Nb alloy, as discussed before, Nb addition enlarged the coarsen rate so that the 1Nb alloy exhibited larger γ′. This is thought to have made the Vickers hardness of the 1Nb alloy smaller than that of the 0Nb ally. However, Nb in Ni-based superalloys substituted onto the Al sub-lattice, inducing an increment in the γ′ anti-phase boundary (APB) energy, which enhanced σ_p_ [15]. One should notice the fact that Nb addition also increased the content of γ′ in the 1Nb alloy, which enhanced the strength. Comprehensively considering the influence of these factors, the experimental results can be reasonably explained.

## 5. Conclusions

In summary, this work concerns the effect of Nb addition on γ′ coarsening and mechanical properties change of Ni-20Co-12Cr PM superalloys during long-term thermal exposure. The kinetics of γ′ coarsening and its relationship with the Vickers hardness at room temperature were discussed. Therefore, the following conclusions can be made:

1. During long-term thermal exposure at 800 °C, the average grain size of both the 0Nb and 1Nb alloys did not change while the γ′ precipitate coarsened visibly. The morphology of γ′ changed from near-spherical to cuboidal shape in both alloys where the 1Nb alloy showed the tendency earlier because Nb addition enlarged the γ/γ′ lattice misfit. With Nb addition, the diffusion coefficients of Al and Nb in the γ matrix increased, resulting a larger coarsening rate in the 1Nb alloy. The γ′ content of the 1Nb alloy is about 4% more than the 0Nb alloy because of Nb addition.
2. The Vickers hardness declined gradually in both alloys with exposure time increasing, which is because the strengthening provided by γ′, including σ_p_ and σ_Oro_, decreased obviously with γ′ coarsening. Based on the compositional and microstructural theoretical calculating, the calculated values σ_y_ are in good agreement with the experimental values $\sigma_{y}^{\exp}$. The precipitate strengthening has the greatest enhancement in the yield strength at room temperature. Solid solution strengthening and grain boundary strengthening, contributing to the yield strength at ambient temperature, were founded to remain unchanged during long-term thermal exposure.

## Figures and Tables

**Table 1:** Compositions of two alloys in weight percent (wt%) tested by inductively coupled plasma optical emission spectroscopy (ICP-OES).

Alloys	Ni	Nb	Al	Ti	Ta	Co	Cr	Mo	W	C	B	Zr	Hf
0Nb	Bal.	-	3.04	3.05	3.99	20.3	12.1	3.06	1.98	0.052	0.012	0.072	0.11
1Nb	Bal.	0.95	3.11	2.92	3.82	20.6	12.5	3.04	2.0	0.07	0.056	0.022	0.18

**Table 2:** The mole fraction (calculated at 800 °C by Thermo-Calc software with the TTNI8 database) and Vegard coefficients of elements in γ and γ′ phase [26]. β_i_ values of elements in γ and γ′ of Ni-based superalloys at room temperature (MPa/at.%^1/2^) [33,34].

Parameters	Ni	Co	Cr	Mo	W	Al	Ti	Ta	Nb	Hf	Zr
0Nb x_i_	0.233349	0.156995	0.126712	0.016906	0.004792	0.011208	0.002071	0.000676	0.000000	0.000005	0.000015
0Nb x′_i_	0.284871	0.042305	0.006538	0.001013	0.001478	0.054483	0.034979	0.011237	0.000000	0.000104	0.000054
1Nb x_i_	0.202726	0.150001	0.124994	0.014903	0.004534	0.009495	0.001539	0.000505	0.000204	0.000006	0.000004
1Nb x′_i_	0.298121	0.046418	0.006645	0.000959	0.001524	0.057644	0.033818	0.010769	0.005393	0.000147	0.000017
V_i_	-	0.0196	0.11	0.478	0.444	0.179	0.422	0.7	0.7	1.031	0.966
V′_i_	0.0126	−0.004	−0.004	0.208	0.194	-	0.258	0.5	0.46	0.777	0.706
β_i_^γ^	-	39.4	337	1015	997	225	775	1191	1183	1401	2359
β_i_^γ′^	-	-	11	41.88	40	-	18.3	78.33	56	159	163.7

**Table 3:** The calculated lattice parameters (Å) at ambient and 800 °C, and misfit *δ* at 800 °C of the 0Nb and 1Nb alloys.

Alloys	a_γ_^RT^	a_γ′_^RT^	a_γ_	a_γ′_	δ
0Nb	3.55460	3.58864	3.61504	3.64173	0.73561%
1Nb	3.55268	3.59075	3.61313	3.64384	0.84632%

**Table 4:** Average grain size (AGS) during long-term exposure at 800 °C.

AGS (μm)	0 h	100 h	200 h	500 h	1000 h
0Nb	20.53	19.84	20.48	20.52	20.57
1Nb	21.99	22.90	22.16	22.85	22.03

## Data Availability

All data are included in the paper.
